# In Vitro Activity of New β-Lactamase Inhibitor Combinations against *bla*_NDM_, *bla*_KPC_, and ESBL-Producing Enterobacteriales Uropathogens

**DOI:** 10.3390/antibiotics12101481

**Published:** 2023-09-25

**Authors:** Lubna Razaq, Fakhur Uddin, Shahzad Ali, Shah Muhammad Abbasi, Muhammad Sohail, Nabila E. Yousif, Hala M. Abo-Dief, Zeinhom M. El-Bahy

**Affiliations:** 1 Department of Microbiology, University of Karachi, Karachi 75270, Pakistan; lubna_qau@yahoo.com; 2 Department of Microbiology, Basic Medical Sciences Institute (BMSI), Jinnah Postgraduate Medical Center (JPMC), Karachi 75510, Pakistan; 3 Department of Urology, Jinnah Postgraduate Medical Center (JPMC), Karachi 75510, Pakistan; shazaid.ali@jpmc.edu.pk; 4 Department of Main Clinical Laboratory, Jinnah Postgraduate Medical Center (JPMC), Karachi 75510, Pakistan; shah.samad@hotmail.com; 5 Department of Science and Technology, University College-Ranyah, Taif University, P.O. Box 11099, Taif 21944, Saudi Arabia; neibrahim@tu.edu.sa (N.E.Y.); h.abodeif@tu.edu.sa (H.M.A.-D.); 6 Department of Chemistry, Faculty of Science, Al-Azhar University, Cairo 11884, Egypt; zeinelbahy@azhar.edu.eg

**Keywords:** meropenem/vaborbactam, ceftazidime/avibactam, *Klebsiella pneumoniae* carbapenemase, New Delhi metallo-β-lactamase

## Abstract

Antibiotic resistance in uropathogens has increased substantially and severely affected treatment of urinary tract infections (UTIs). Lately, some new formulations, including meropenem/vaborbactam (MEV), ceftazidime/avibactam (CZA), and ceftolozane/tazobactam (C/T) have been introduced to treat infections caused by drug-resistant pathogens. This study was designed to screen Enterobacteriales isolates from UTI patients and to assess their antimicrobial resistance pattern, particularly against the mentioned (new) antibiotics. Phenotypic screening of extended-spectrum β-lactamase (ESBL) and carbapenem resistance was followed by inhibitor-based assays to detect *K. pneumoniae* carbapenemase (KPC), metallo-β-lactamase (MBL), and class D oxacillinases (OXA). Among 289 Enterobacteriales, *E. coli* (66.4%) was the most predominant pathogen, followed by *K. pneumoniae* (13.8%) and *P. mirabilis* (8.3%). The isolates showed higher resistance to penicillins and cephalosporins (70–87%) than to non-β-lactam antimicrobials (33.2–41.5%). NDM production was a common feature among carbapenem-resistant (CR) isolates, followed by KPC and OXA. ESBL producers were susceptible to the tested new antibiotics, but NDM-positive isolates appeared resistant to these combinations. KPC-producers showed resistance to only C/T. ESBLs and carbapenemase encoding genes were located on plasmids and most of the genes were successfully transferred to recipient cells. This study revealed that MEV and CZA had significant activity against ESBL and KPC producers.

## 1. Introduction

Urinary tract infections (UTIs) are among the most prevalent bacterial infections in communities. UTI and its related complications cause about 150 million deaths annually at the global level [1,2]. Members of the Enterobacteriales family are widespread pathogens, amongst which species of Enterobacteriaceae (*Escherichia coli*, *Enterobacter*, *Klebsiella pneumoniae*, and *Citrobacter*) and Morganellaceae (*Morganella*, *Proteus*, and *Providencia*) are the common causes of community- and hospital-acquired UTIs [3,4]. The burden of infections caused by extended-spectrum β-lactamase (ESBL) producers and/or carbapenem-resistant Enterobacteriales (CRE) results in an increase in hospital stays, comorbidities, treatment costs, and mortality compared with infections caused by carbapenem-susceptible Enterobacteriales [5]. The higher prevalence of multidrug-resistant (MDR) and carbapenem-resistant (CR) uropathogens limits common and oral treatment options [6]. The prevalence of extended-spectrum β-lactamase-producing (ESBL) Enterobacteriales has increased to 75% in some parts of the world [7].

Non-β-lactam antimicrobial agents and carbapenems are treatment options for ESBL-producing Enterobacteriales species. However, the coexistence of resistance to non-β-lactam antimicrobial agents and carbapenems in ESBL producers further restricts available treatment options [8]. In Enterobacteriales isolates, carbapenemase production is the main mode of resistance to carbapenems. The resistant genes *(bla*_TEM_, *bla*_SHV_, *bla*_CTX-M_, *bla*_KPC_, *bla*_NDM_, *bla*_OXA-48_, etc.) are frequently found on mobile genetic elements (plasmids, integrons, insertion sequences, and transposons) and have the potential to be widespread among bacteria, and this has become a major global health threat [9].

The prevalence of a particular type of carbapenemase in CRE varies from region to region. For example, in Asia, New Delhi metallo-β-lactamase (NDM-1) is commonly found, whereas *Klebsiella pneumoniae* carbapenemase (KPC) is more prevalent in Europe and the US [10]. CRE cause severe infections and hospitalization, along with a high mortality of 40–56% with KPC-producing *K. pneumoniae* and up to 88% with NDM-1 producing Enterobacteriales [11]. It is reported that the multidrug-resistant (MDR) and extensively drug-resistant (XDR) bacteria are mainly ESBLs and/or carbapenemase producers. To overcome the problem of antimicrobial resistance, new β-lactamase inhibitors are required to treat infections caused by resistant pathogens. The Food and Drug Authority (FDA, USA) approved ceftolozane/tazobactam (C/T) for ESBL producers and ceftazidime/avibactam (CZA) and meropenem/vaborbactam (MEV) for KPC and/or ESBL-producing bacteria [12]. Ceftolozane in combination with a β-lactamase inhibitor (tazobactam) is a well-known combination of fifth generation cephalosporin and is active against ESBL producers. C/T has good activity against selected members of class A ESBLs, including TEM, SHV, and CTX-M, and a few members of class C (AmpCs) and class D, but is inactive against Class A and B carbapenemases [13]. MEV is a new β-lactamase inhibitor combination that has a spectrum similar to CZA and is ineffective against MBLs. Vaborbactam has less inhibitory activity against class D carbapenemases (OXA-48) than against class A enzymes [14]. Nonetheless, the efficacy of these new β-lactam/β-lactamase inhibitor combinations is reportedly affected by non-enzymatic resistance mechanisms, such as outer membrane protein (OMP) or alterations in penicillin binding protein (PBP) and overexpression of efflux systems [15].

These antibiotics have not been marketed in Pakistan, therefore their efficacy data are not available, particularly for resistant bacteria. It has been hypothesized that mutations and resistance can be detected prior to the use of a drug; hence, baseline data are required for such newly introduced combinations. In this study, we evaluated the in vitro activity of ceftazidime/avibactam, ceftolozane/tazobactam, and meropenem/vaborbactam against carbapenem-resistant and ESBL-producing isolates from patients with UTI.

## 2. Results

During the study period, a total of 289 Enterobacteriales were isolated. The majority of isolates were from outpatients (151, 52.25%), followed by inpatients (138, 47.75%). The female preponderance was common overall (187/289, 64.70%) and in both groups as outpatients (115, 76.15%) and in admitted UTI patients (72, 52.17%). Most patients were aged between 15 and 35 years (180, 62.28%), followed by 36–60 years (109, 37.72%), in both community and admitted patients. The antibiotic prescription data from OPD and the urology ward were collected. Third and fourth generation cephalosporins (40%) were the most prescribed antibiotics, followed by quinolones (20%) and piperacillin/tazobactam (10%). Less frequently prescribed antibiotics included aminoglycosides, nitrofurantoin, and fosfomycin, whereas imipenem and meropenem were found to be only prescribed in wards (15%).

### 2.1. Frequency of Enterobacteriales Species and Antimicrobial Susceptibility

**Figure 1:**
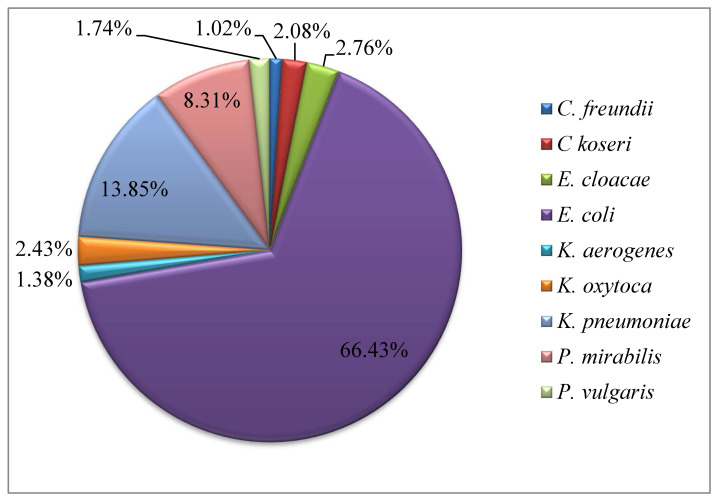
Prevalence of Enterobacteriales species in UTI patients.

Of the 289 Enterobacteriales isolates, *Escherichia coli* (192, 66.43%), *K. pneumoniae* (40, 13.8%), and *Proteus mirabilis* (24, 8.31%) were the most common uropathogens (Figure 1). Most isolates were resistant to ampicillin, cefazolin, cefuroxime (252, 87.2%), and trimethoprim (227, 78.5%). Overall resistance to carbapenems was 34.25% (99 isolates). Non-β-lactam antimicrobial agents exhibited promising activities, with a lower resistance in uropathogens (28.3–37.7%) against amikacin, fosfomycin, and nitrofurantoin in comparison to penicillins and cephalosporins (Table 1).

### 2.2. Comparison of Antimicrobial Resistance Pattern in OPD and Ward Patients

The antibiotic resistance patterns against antimicrobial agents between OPD and hospitalized uropathogens were analyzed. The resistance to antimicrobials was higher in uropathogens isolated from inpatients compared with OPD patient isolates, except for amoxicillin, ceftaroline, and aztreonam. This difference was statistically significant (*p* < 0.05). Cefazolin, cefuroxime, and piperacillin showed the highest resistance rates among the hospitalized patients (132 (95.65%)). In contrast, cefoxitin, ceftaroline, cefepime, ceftazidime, and ceftriaxone ranged from 96 (69.56%) to 120 (86.95%) in admitted patients and from 147 (54.3%) to 220 (76.1%) in OPD patients (Table 2).

### 2.3. Phenotypic and Genotypic Detection of ESBL Producers and Carbapenem-Resistant (CR) Enterobacteriales

Phenotypic characterization revealed that out of the 289 Enterobacteriales isolates, 125 (43.25%) were ESBL producers. The frequency of ESBL producers in admitted patients was high (70/125, 56%) compared with outpatients. The frequency of ESBL production was higher in *E. cloacae* (6/8, 75%), *K. oxytoca* (4/7, 57.14%), and *K. aerogenes* (2/4, 50%) but moderate in *E. coli* (87/192, 45.31%), *K. pneumoniae* (16/40, 40%), and *P. mirabilis* (7/24, 29.16%). The phenotypically positive isolates were subjected to molecular characterization of ESBLs. PCR results revealed *bla*_SHV_ (70/125, 56%) as the most common ESBL type, followed by *bla*_TEM_ (50/125, 40%) and *bla*_CTX-M_ (5/125, 4%).

**Figure 2:**
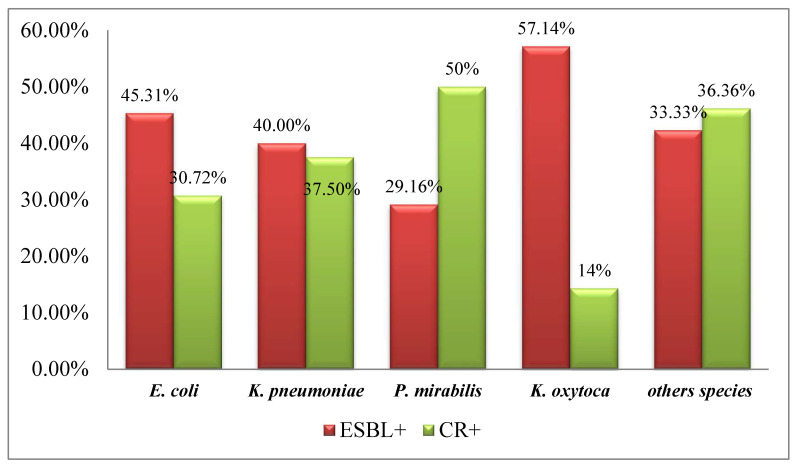
Prevalence of ESBL and carbapenem-resistant Enterobacteriales isolates.

Overall resistance to carbapenems was (99/289, 34.25%). The prevalence of carbapenem-resistant isolates was higher in ward patients (92/99, 92.92%) in comparison with OPD. Phenotypically (by combined disc diffusion method), a prevalence of MBL, OXA, and KPC producers was found at levels of 81.81%, 13.13%, and 5.05%, respectively (Figure S1). Carbapenem resistance was higher (50–66%) in *P. vulgaris*, *P. mirabilis*, *C. koseri,* and *K. aerogenes*, whereas in other species the prevalence was >40% (Figure 2).

Of the 99 CR isolates, 81 were found to have *bla*_NDM_ (81.81%), 13 had *bla*_OXA-48_ type (13.13%), and 5 carried *bla*_KPC_ (5.05%). *bla*_VIM_ and *bla*_IMP_ genes were not detected in any isolates (Figures S2 and S3). The coexistence of different ESBLs and ESBLs with carbapenemases was very high (110/224, 49.10%). In 37.03% of the isolates, *bla*_SHV_ coexisted with *bla*_NDM_ and in 24.1% with *bla*_TEM_. The coexistence of *bla*_NDM_ with *bla*_TEM_ was much lower (16.04%), while *bla*_OXA-48_ appeared more frequently (53.84%) than *bla*_CTX-M_. There was no coexistence of the tested markers in *bla*_KPC_-positive isolates.

### 2.4. Plasmid Analysis

According to the PCR results of plasmid analysis, 98.4% (123/125) of the ESBL genes and 100% (99/99) of the CR encoding genes were plasmid-mediated. To assess the transferability of the plasmid-mediated resistance genes, conjugation experiments were conducted on carbapenem-resistant (*bla*_NDM_, *bla*_OXA-48_, and *bla*_KPC_) and ESBL and *bla*_SHV_, *bla*_TEM_, and *bla*_CTX-M_ producers. The results of conjugation showed that out of 81 *bla*_NDM_-carrying isolates, 79 (97.53%) isolates successfully transferred the *bla*_NDM_ gene to the recipient, whereas the transfer rate was higher in the case of *bla*_OXA-48_ and *bla*_KPC_ (13/13, 5/5; 100%, respectively). Analysis of the plasmid DNA isolated from the recipient ESBL producers (transconjugants) revealed that *bla*_TEM_ (47/50; 94%), *bla*_SHV_ (51/70; 72.85%), and *bla*_CTXM_ (5/5; 100%) were transferred to the recipient.

Plasmid transformation was successful in all carbapenem-resistant and ESBL-producing isolates carrying *bla*_NDM_ (81/81), *bla*_OXA-48_ (13/13), *bla*_KPC_ (5/5), *bla*_TEM_ (50/50; 100%), *bla*_SHV_ (68/70; 97.14%), or *bla*_CTX-M_ (5/5; 100%).

### 2.5. Susceptibility Patterns to Newer Drugs

The overall susceptibility pattern of Enterobacteriales to meropenem/vaborbactam revealed that 60.67% of the isolates were susceptible (Table 3). All ESBL producers (125 isolates) without the coexistence of carbapenemases (MBL or OXA) were found to be susceptible to meropenem/vaborbactam, with MIC values ranging from 0.04 to 2 mg L^−1^. All KPC-positive isolates (5/99; 5.05%) were sensitive to meropenem/vaborbactam, with MICs ranging from 0.5 to 2 mg L^−1^, whereas all *bla*_NDM_-harboring variants (33) with the coexistence of OXA-48 (4) and ESBL (44) exhibited resistance, with MIC values from 16 to 64 mg L^−1^. Of the 13 *bla*_OXA-48_ producers, only 4 appeared susceptible to meropenem/vaborbactam (MIC ranged from 2 to 8 mg L^−1^).

The majority of isolates (191; 66.08%) were susceptible to CZA. All ESBL producers and carbapenem-susceptible isolates were sensitive to this combination, with MICs ranging from 0.016 to 1 mg L^−1^. All NDM and KPC producers exhibited resistance to CZA (MIC 8–64 mg L^−1^). Out of 13 *bla*_OXA-48_ positive isolates, 11 showed resistance to CZA, with MIC values of 8–16 mg L^−1^.

Ceftolozane/tazobactam was active against all ESBL producers, with MICs of 0.016–1 mg L^−1^. All carbapenemase producers, including *bla*_OXA-48-_, *bla*_KPC-_, and *bla*_NDM_-harboring isolates, showed resistance to this drug. The MIC of C/T was higher (32–256 mg L^−1^) for NDM producers than for OXA-48 or KPC producers (16–32 mg L^−1^).

### 2.6. Prevalence of Multi-Drug-Resistant (MDR), Extensively Drug-Resistant (XDR), and Pan Drug-Resistant (PDR) Isolates

**Figure 3:**
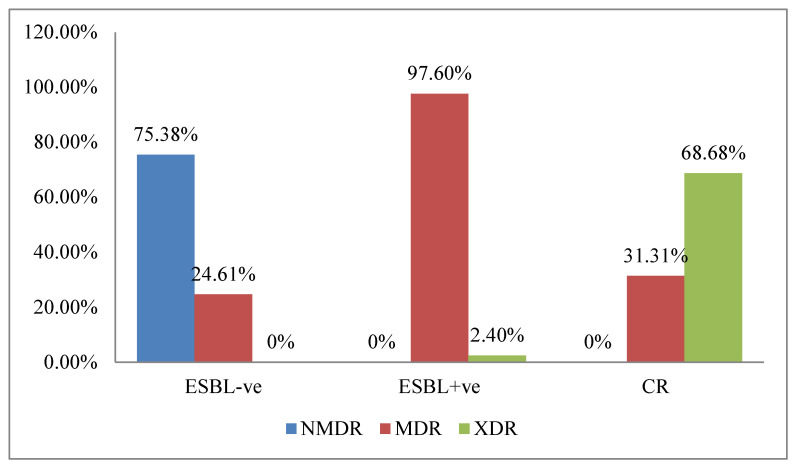
Prevalence of multi-drug-resistant (MDR) and extensively drug-resistant (XDR) isolates in relation with ESBLs and/or carbapenem-resistant isolate production (*n* = 289).

Of the 289 isolates, 69 (58.5%) were MDR and 71 (24.6%) were XDR. The majority of MDR isolates (122/125; 97.6%) were ESBL producers. Most carbapenem-resistant isolates belonged to the XDR category (68/99, 68.68%). None of the isolates were found to be PDR (Figure 3).

The susceptibility of non-β-lactam antimicrobial agents was analyzed for its association with ESBL or carbapenem resistance using the chi-square test. The isolates were categorized into four groups: Group 1, non-ESBL producers; Group 2, ESBL producers; Group 3, carbapenem-susceptible (CS); and Group 4, carbapenem-resistant (CR) isolates. Resistance to quinolones, aminoglycosides, fosfomycin, and nitrofurantoin was higher in ESBL producers than in non-ESBL producers and similarly was higher in CR than in CS. The difference in susceptibility was statistically significant at *p* < 0.0001 (Table 4).

## 3. Materials and Methods

### 3.1. Study Setting and Period

This study was conducted during the period 2 June 2021 to 30 November 2021 in the department of Microbiology, University of Karachi in collaboration with the departments of Urology and Microbiology, Jinnah postgraduate medical center (JPMC), Karachi.

### 3.2. Sample Collection and Processing

The midstream urine samples were collected from ward and OPD patients. Patients in ICU prescribed with antibiotics and/or catheterized were excluded. A total of 289 Enterobacteriales strains were isolated from the urine samples of UTI patients using a cysteine lactose electrolyte deficient (CLED) medium (Oxoid, Hampshire, UK). The isolates were initially identified by traditional methods, including Gram staining, growth characteristics, and biochemical tests [16]. Identification was confirmed using API 20E (bioMérieux SA, Marcy-I’Etoile, France). Antimicrobial susceptibility testing was performed according to CLSI guidelines (2021). *E. coli* (ATCC 25922) and *Pseudomonas aeruginosa* (ATCC 27853) strains were used as quality control strains for the antimicrobial susceptibility testing.

### 3.3. Phenotypic Detection of Extended Spectrum β-Lactamases (ESBLs) and Carbapenemases

Screening for ESBL production was based on resistance to one or more 3rd or 4th generation cephalosporin or aztreonam [17]. Isolates resistant to one or more oxyimino-β-lactam, such as cefotaxime, ceftazidime, or aztreonam, were considered as subgroup to be β-lactamases producers [18]. The screened ESBL producers were phenotypically confirmed using the double-disc synergy test as described previously. The method was performed using a combination of cefepime (30 μg), ceftazidime (30 μg), ceftriaxone (30 μg), aztreonam, and amoxicillin/clavulanate (20/10 μg). After incubation, ESBL producers were identified by considering an expansion of the zone of inhibition due to third generation cephalosporins or aztreonam towards amoxicillin/clavulanate.

The carbapenem-resistant (CR) isolates were analyzed for carbapenemases, including OXA, MBL, and KPC, using an inhibition-based assay with EDTA and phenylboronic acid (PBA) in combination with a temocillin disc (30 µg). After making a bacterial lawn on Mueller Hinton agar (MHA) plates, discs of imipenem (IMP, 10 µg), meropenem (MEM, 10 µg), and temocillin (30 µg) were placed. One set of IMP and MEM discs was impregnated with 10 µL (0.5 µg) of EDTA solution; the second set of IMP and MEM discs was added to 20 µL (400 µg) of PBA; the third set of these discs was used without EDTA or PBA [19]. After incubation at 37 °C for 24 h, the zones of inhibition were observed. An increase of ≥5 mm in the zone of inhibition due to imipenem or meropenem with PBA and ≥7 mm with EDTA was interpreted as KPC or MBL producers, respectively. CRE isolates that were found to be negative for MBL and KPC but resistant (zone of inhibition ≤ 11 mm) to temocillin were interpreted as OXA-48 type producers [20].

### 3.4. Genotypic Detection of Extended Spectrum β-Lactamases (ESBLs) and Carbapenemase

Phenotypically screened ESBL producers were investigated for the presence of common genotypic ESBLs variants, including *bla*_TEM_, *bla*_SHV_, and *bla*_CTX-M_. The CRE isolates were studied for the presence of carbapenemase-encoding genes (Table S1).

DNA extraction was performed using a kit method (WizPrep^TM^ DNA Purification Mini Kit; Wizbiosolutions; Gyeonggi-do, Republic of Korea). PCR amplification was performed using a thermal cycler (Kryatec SC300G-R2; Kyratec, Queensland, Australia). The reaction mixture (20 μL) contained 2 μL DNA template, 10 μL (DreamTaq DNA polymerase; Thermo Fisher Scientific Baltics, Vilnius, Lithuania), 0.5 μL of reverse and 0.5 μL of forward primers, and 7 μL DNase free water. The primer sequences, annealing temperatures, and product sizes are listed in Table 1. PCR conditions included initial denaturation at 94 °C for 4 min, denaturation at 94 °C for 1 min, annealing at different temperatures according to the primer for 30 s, extension at 72 °C for 1 min, and final extension at 72 °C for 10 min. PCR amplification was performed for 35 cycles. The PCR products were separated on 2% agarose gel. *K. pneumoniae* (ATCC 700603) and *E. coli* (ATCC 35218) were used as positive control for *bla*_SHV_ and *bla*_TEM_.

### 3.5. Plasmid Analysis

Plasmid DNA was extracted from the PCR-positive isolates for carbapenemases and ESBLs. Plasmid DNA was extracted using a GeneJET plasmid mini kit (Thermo Fisher Scientific Baltics, Vilnius, Lithuania) and PCR was performed using the same primers and conditions as described in the previous section (genotypic detection of ESBL and carbapenemases).

### 3.6. Conjugation and Transformation Assays

The transferability of ESBLs (*bla*_SHV_, *bla*_TEM_, and *bla*_CTX-M_) and carbapenem-resistance genes (*bla*_NDM_, *bla*_KPC_ and *bla*_OXA-48_) was determined using sodium azide-resistant *E. coli* J53 as the recipient in conjugation experiments. The recipient strain of *E. coli* J53 and the donor strains were separately cultivated in brain heart infusion broth (BHI) for 24 h at 37 °C and inoculum was adjusted to a 0.5 McFarland standard. The donor strain (30 µL) and the recipient strain of *E. coli* J53 (90 µL) were applied on a microporous membrane. To recover the transconjugants, the conjugation solutions were subsequently diluted and plated onto BHI agar plates supplemented with sodium azide (200 mg L^−1^) and meropenem (2 mg L^−1^). PCR and antimicrobial sensitivity tests helped to ascertain that the transconjugants were descendants of the recipient strain [20].

Ae transformation assay was also performed to investigate transferability of resistance genes by preparing the recipient cells of *E. coli* and *K. pneumoniae*. Briefly, bacterial suspensions of the sensitive isolates in Luria–Bertani (LB) broth were prepared. The turbidity was adjusted to 5 × 10^7^ cells mL^−1^, and pellets were collected by centrifugation at 4 °C at 4000 rpm for 10 min. Pellets were suspended in 20 mL ice-cold 0.1 M CaCl_2_ solution and incubated on ice for 30 min. Competent cells were obtained by centrifugation at 4 °C at 4000 rpm for 10 min and the supernatant was discarded. Competent cells were suspended in 5 mL ice-cold 0.1 M CaCl_2_ with 15% glycerol and stored at −80 °C for transformation. For the transformation assay, 5 µL of plasmid extract was added to 50 µL competent cell aliquots, incubated on ice for 30 min, transferred to a 42 °C water bath for 30 s, and again incubated on ice for 2 min. One mL of LB broth was added and tubes were incubated in a shaking incubator at 37 °C and 200 rpm for 1 h [21]. Inoculums were transferred to the appropriate antibiotic-containing media plates and incubated at 37 °C for 24 h and the results were recorded.

### 3.7. Susceptibility of ESBL Producers and CRE Isolates to Newer β-Lactam/β-Lactamase Inhibitor Combinations

Meropenem/vaborbactam, ceftolozane/tazobactam (C/T), and ceftazidime/avibactam (CZA) was purchased from Hardy diagnostics, Santa Maria, CA, USA. The susceptibility of the isolates to meropenem/vaborbactam (20/10 µg), C/T (30/10 µg), and CZA (30/20 µg) was tested in accordance with CLSI guidelines (CLSI 2021), whereas the MICs were determined using Estrips (bioMerieux SA, Marcy-l’Étoile, France). Enterobacteriales isolates were classified into different resistance categories (MDR, XDR, and PDR) as defined earlier [22].

### 3.8. Statistical Analysis

The data were exported from Excel to SPSS version 22 and analyzed for descriptive statistics, including frequencies and percentages. The associations between variables with appropriate statistical tests were determined at *p* < 0.05.

## 4. Discussion

Antimicrobial resistance (AMR), particularly by ESBL producers and CRE, poses a serious threat to the health sector worldwide. It is estimated that AMR will lead to 10 million deaths annually by 2050. One major challenge in the prevention and control of AMR is the determination of the actual burden and type of resistance mechanism, especially in countries where AMR surveillance is not integrated because of the scarcity of authentic data [19]. In our study, Enterobacteriales displayed high resistance to cefazolin (87.1%), ceftriaxone (76.1%), trimethoprim/sulfamethoxazole (74.3%), cefepime (70.6%), and ciprofloxacin (68.8%), whereas resistance to meropenem (28.3%), imipenem (34.25%), amikacin (35.6%), and nitrofurantoin (41.5%) was lower. In the present study setting, most prescribed antibiotics were third generation cephalosporins, there antibiotic pressure is reflected in the antibiotic resistance pattern (87%) against this class of antibiotics. The resistance pattern of third generation cephalosporins was higher than those of meropenem and imipenem. These results are consistent with the previous studies [23,24]. The resistance patterns of both groups (ward and OPD) of the isolates showed higher resistance to penicillin, cephalosporins, and quinolones. This is consistent with the results of a previous study [25].

The occurrence of ESBL-producing strains varies in different geographical regions and even differs between hospitals, depending on antibiotic pressure and other risk factors. The reported prevalence of ESBL-producing Enterobacteriales varies from 26% to 66.2% across different regions [26,27]. Therefore, regional surveillance programs and their continuity are an essential part of the control and hospital antibiotic policies. In the present study, the prevalence of ESBL producers was 46.71%, which contradicts the findings of Nasir et al. (2021) [26], where a higher (66.2%) prevalence of ESBL-producing uropathogens was reported. Molecular detection of the genes showed that 30% of *bla*_NDM_-carrying isolates also harbored *bla*_SHV_, while 53.84% *bla*_OXA-48_ also harbored *bla*_CTX-M_. An earlier finding also reported the coexistence of carbapenemases and β-lactamases [28]. The prevalence of carbapenem-resistant isolates in the present study was higher (34.25%) than that reported (5%) by Nasir et al. (2021) [26]. The lower prevalence of carbapenem-resistant isolates reported by Nasir et al. (2021) [26] can be attributed to the small sample size, which was skewed towards *E. coli* uropathogens.

It is generally known that the majority of CRE isolates carry genes encoding carbapenemases, especially KPCs, NDM, and OXA-48-like enzymes; the widespread presence of these markers has been reported globally [29]. NDM production is a common mechanism in CRE from South Asia [30], which was also affirmed in a recent study from Pakistan showing an 82.69% prevalence among CRE [31]. The present study reaffirmed these findings that NDM is the most common carbapenemase among carbapenemase-producing Enterobacteriales, followed by *bla*_OXA-48_. In studies that have determined the global prevalence of CPE isolates, OXA-48 types have been reported to be the second or third most common genetic determinant in CPE isolates [29].

It has been widely reported that the spread of antimicrobial resistance is primarily caused by the dissemination of large plasmids carrying multiple antibiotic resistance genes. Antibiotic-resistance genes, such as CR-encoding genes, are plasmid-mediated and often coharbored with different antibiotic resistance markers, such as ESBL-encoding genes. In this study, the analysis of plasmid showed that all CR genes, i.e., *bla*_NDM_, *bla*_OXA-48,_ and *bla*_KPC_ (100%), and ESBL (98.4%) genes were plasmid-mediated; this finding was in corroboration with a previous study [32]

The spread of the *bla*_OXA-48_ gene was likely facilitated by the transfer of plasmids. Based on conjugation experiments, the *bla*_OXA-48_-carrying plasmid identified here is capable of interspecies conjugation. There is a 10-fold higher conjugation efficiency of the plasmid carrying *bla*_OXA-48_ compared with that of *bla*_KPC-3_-carrying genes [33,34]. To assess the transferability of plasmids and resistance genes, conjugation experiments were conducted on carbapenem-resistant isolates and ESBL producers. The results showed that carbapenem-resistant Enterobacteriales conjugated 97.53% in *bla*_NDM_ positive Enterobacteriales, corroborating findings of Adler et al. and Song et al. [35,36]. Plasmid transformation occurred successfully in all carbapenem-resistant and ESBL-producing isolates. In this study, carbapenemases including *bla*_NDM_, *bla*_OXA-48_, and *bla*_KPC_ (100%) were successfully transformed from resistant Enterobacteriales to sensitive isolates, reflecting the high risk of horizontal resistance transfer. Similar results were reported by Baraka et al., who successfully transferred CR genes in *E. coli* and *K. pneumoniae* [21]. Our experimental data showed a high transferability rate for *bla*_OXA-48_ (100%). A study conducted by Elshamy et al. in Egypt revealed a similar trend in the transferability rate of *bla*_OXA-48_ among their clinical isolates, indicating the widespread dissemination of this resistance gene in different regions [37].

The coexistence of ESBL and/or carbapenemase production with resistance to non-β-lactam antimicrobial agents (fluoroquinolones, aminoglycosides, and trimethoprim/sulfamethoxazole) further reduces treatment options [38] and hence poses a great threat to the public health sector. Recently, meropenem/vaborbactam, CZA, and C/T have been approved as alternative treatment options for such resistant bugs [39]. The current study summarizes the in vitro antimicrobial susceptibility of these drugs against ESBLs, KPC, and MBLs and other CRE uropathogens. These antimicrobial agents are currently not readily available in Pakistan, hence their use is limited. The findings of the present study revealed that all the ESBL- (*bla*_SHV_, *bla*_TEM_,and *bla*_CTX-M-4_) and KPC-producing uropathogens were susceptible to meropenem/vaborbactam, whereas all *bla*_NDM_ producers and *bla*_OXA-48_ producers were resistant to this regimen. This susceptibility pattern is in line with previous reports by Dhillon et al. (2018), Shortridge et al. (2021), Yahav et al. (2021),and Bakthavatchalam et al. (2022) [14,40,41,42], where the overall susceptibility of ESBL-producing uropathogens to CZA was found to be promising, but NDM or KPC producers were resistant. OXA-48 producers were less susceptible (33.33%) to CZA, which is in line with a recent report [43]. The isolates carrying both OXA and CTX-M were resistant to CZA, however only OXA-harboring isolates were susceptible to this antibiotic. This limits the usefulness of CZA monotherapy for infections caused by *bla*_CTX-M-_ and *bla*_OXA-48_-carrying isolates [44]. Emerging resistance to CZA in an isolate with an OXA-48 enzyme was associated with Pro170Ser and Thr264Ile substitutions in a coproduced CTX-M-14 ESBL, without changes to the OXA-48 marker (Both et al. 2017) [44]. In contrast, Mavroidi et al. (2020) reported that isolates carrying OXA + CTX-M were more susceptible to this combination [45]. Among the OXA-48-positive isolates, the coexistence of ESBL also reduced susceptibility to MEV, as reported earlier that the presence of ESBLs and outer membrane protein (Opm) alterations are associated with reduced susceptibility [46]. An earlier study presented the combination of aztreonam/ceftazidime/avibactam as the best treatment option for MBL production [44]. The susceptibility of Enterobacteriales to C/T was in line with Karlowsky et al. (2020), as most of the ESBL-producing Enterobacteriales (98.4%) were susceptible, but all CRE isolates were resistant to this combination [47]. Hence, minimizing the frequent use of carbapenems is a good option for the treatment of ESBL-producing Enterobacteriales.

## 5. Conclusions

In conclusion, we report that meropenem/vaborbactam, ceftolozane/tazobactam, and ceftazidime/avibactam have significant activity against ESBL-producing uropathogens. The resistance to β-lactam antibiotics was higher in comparison with the non-β-lactams, except the carbapenems. The carbapenem-resistant isolates were mostly NDM producers, which were resistant to the new β-lactamase inhibitor combinations. Meropenem/vaborbactam and ceftazidime/avibactam were active against *bla*_OXA-48_ and *bla*_KPC_. However, this study has limitations in analyzing a small sample size of KPC and OXA-producing isolates; hence, large-scale molecular studies are required for the exploration of the different resistance mechanisms other than the ESBL and carbapenemases, which will accurately provide the effectiveness of these antibiotics. Moreover, continuous monitoring and surveillance are required to better manage the issues caused by the rapid emergence of antimicrobial resistance.

## 6. Limitation of Study

We conducted a study at a single center, which may limit the generalization of our findings to a broader population. However, since the tertiary care hospital caters to the need of a vast population from various cities in the county, our study still provides valuable information about the uropathogens commonly found in our clinical setting, which can serve as a starting point for further research across multiple centers. Our sample size was relatively small compared with data from global surveillance systems. This is because we focused on patients with urinary tract infections in our institution during the study period. To address this limitation, future studies should aim to replicate our research in diverse clinical settings with larger sample sizes, encompassing a broader population of patients with urinary tract infections. Our goal was to clarify the scope of our research and encourage future studies to expand upon our work. These findings provide important insights into the characteristics of the uropathogens responsible for hospital-acquired infections in a specific context.

## Figures and Tables

**Table 1:** Antimicrobial resistance patterns of Enterobacteriales isolates from urine samples (*n* = 289).

Antibiotics	*E. coli* *n* = 192 (%)	*K. pneumoniae* *n* = 40 (%)	*P. mirabilis* *n* = 24 (%)	Other Species * n = 33 (%)	Total 289 (%)
Cefazolin	163 (84.8)	35 (87.5)	23 (95.8)	31 (93.9)	252 (87.1)
Cefuroxime	163 (84.8)	35 (87.5)	23 (95.8)	31 (93.9)	252 (87.1)
Ampicillin	163 (84.8)	35 (87.5)	23 (95.8)	29 (87.8)	250 (86.5)
Piperacillin	163 (84.8)	35 (87.5)	23 (95.8)	31 (93.9)	252 (87.2)
Amoxicillin/clavulanic acid	162 (84.8)	35 (87.5)	23 (95.8)	29 (87.8)	249 (85.1)
Piperacillin/tazobactam	156 (81.3)	33 (82.5)	22 (91.6)	29 (87.8)	240 (59.8)
Ceftaroline	126 (65)	29 (72.5)	15 (62.5)	23 (69.6)	193 (66.8)
Cefepime	133 (69.7)	30 (75)	16 (66.6)	25 (75.7)	204 (70.6)
Cefoxitin	95 (49.4)	22 (55)	16 (66.6)	16 (48.4)	147 (54.3)
Ceftriaxone	142 (73.9)	31 (77.5)	19 (79.1)	28 (84.8)	220 (76.1)
Ceftazidime	136 (70.9)	31 (77.5)	16 (66.6)	27 (81.8)	210 (72.6)
Aztreonam	133 (70.3)	30 (75)	16 (66.6)	26 (78.7)	205 (71.6)
Meropenem	48 (25)	12 (30)	10 (41.6)	12 (36.3)	82 (28.3)
Imipenem	58 (30.2)	16 (37.5)	12 (50)	13 (39.3)	99 (34.25)
Amikacin	56 (29.1)	19 (47.5)	13 (54.1)	15 (45.4)	103 (35.6)
Gentamicin	99 (51.5)	24 (60)	15 (62.5)	22 (66.6)	160 (55.3)
Nalidixic acid	135 (70.3)	30 (75)	15 (62.3)	24 (72.7)	204 (70.5)
Norfloxacine	134 (69.7)	29 (72.5)	15 (62.5)	23 (69.6)	201 (69.5)
Ciprofloxacin	129 (67.1)	29 (72.5)	17 (70.8)	24 (72.7)	199 (68.8)
Fosfomycin	61 (31.7)	-	-	-	-
Trimethoprim/sulfamethoxazole	146 (76)	28 (70)	19 (79.1)	23 (69.6)	215 (74.3)
Nitrofurantoin	64 (33.3)	17 (42.5)	24 (100)	15 (45.4)	120 (41.5)
Trimethoprim	151 (78.6)	28 (70)	23 (95.8)	25 (75.7)	227 (78.5)
Tetracycline	124 (64.5)	31 (77.5)	24 (100)	23 (69.6)	202 (69.8)

* Other species included *K. oxytoca* (7), *P. vulgaris* (5), *K. aerogenes* (4), *E. cloacae* (8) *C. freundii* (3), *C. koseri* (6).

**Table 2:** Comparison of antimicrobial resistance patterns in OPD and ward patients.

Antibiotics	Ward Patients 138 (%)	OPD Patients 151 (%)	Total 289 (%)	*Z* Value	*p* Value *
Cefazolin	132 (95.65%)	120 (79.47%)	252 (87.1)	4.1125	<0.00001
Cefuroxime	132 (95.65%)	120 (79.47%)	252 (87.1)	4.1125	<0.00001
Ampicillin	130 (94.20%)	120 (79.47%)	250 (86.5)	3.6615	0.00026
Piperacillin	132 (95.65%)	120 (79.47%)	252 (87.2)	4.1125	<0.00001
Amoxicillin/clavulanic acid	122 (88.40%)	127 (84.10%)	249 (85.1)	1.0573	0.28914
Piperacillin/tazobactam	125 (90.57%)	115 (76.15%)	240 (59.8)	3.2633	0.00112
Ceftaroline	98 (71.01%)	95 (62.91%)	193 (66.8)	1.4604	0.1443
Cefepime	111 (83.43%)	93 (61.58%)	204 (70.6)	3.512	0.00044
Cefoxitin	96 (69.56%)	51 (33.77%)	147 (54.3)	6.0791	<0.00001
Ceftriaxone	120 (86.95%)	100 (66.22%)	220 (76.1)	4.1292	<0.00001
Ceftazidime	111 (83.43%)	99 (65.56%)	210 (72.6)	2.8335	0.00466
Aztreonam	104 (75.36%)	101 (66.89%)	205 (71.6)	1.5849	0.1141
Meropenem	80 (57.97%)	2 (1.32%)	82 (28.3)	10.6698	<0.00001
Imipenem	92 (92.92%)	7 (4.63%)	99 (34.25)	11.0992	<0.00001
Amikacin	83 (60.14%)	20 (13.24%)	103 (35.6)	8.3152	<0.00001
Gentamicin	86 (62.31%)	74 (49.0%)	160 (55.3)	2.0509	0.04036
Nalidixic acid	123 (89.13%)	85 (56.29%)	204 (70.5)	6.2086	<0.00001
Norfloxacine	101 (73.18%)	100 (66.2%)	201 (69.5)	1.2848	0.20054
Ciprofloxacin	100 (72.4%)	99 (65.56%)	199 (68.8)	1.2654	0.20408
Fosfomycin	42 (30.43%)	19 (12.59%)	-	3.7148	0.0002
Trimethoprim/sulfamethoxazole	128 (92.75%)	87 (56.61%)	215 (74.3)	6.8362	<0.00001
Nitrofurantoin	86 (62.31%)	34 (22.51%)	120 (41.5)	6.8588	<0.00001
Trimethoprim	120 (86.95%)	107 (70.86%)	227 (78.5)	3.3295	<0.00086
Tetracycline	125 (90.57%)	77 (50.99%)	202 (69.8)	7.328	<0.00001

* The result is significant at *p* < 0.05.

**Table 3:** Susceptibility patterns of ESBLs and/or carbapenemase producers to newer drugs (meropenem/vaborbactam, MEV, ceftazidime/avibactam (CZA), and ceftolozane/tazobactam (C/T).

Genotypic Resistant Isolates	Susceptibility Pattern					
	MEV *n* (%)		CAZ		C/T	
	S	R	S	R	S	R
ESBL-types (71)	71 (100)	-	71 (100)	-	71 (100)	-
Coexistence ESBL + ESBLs (54)	54 (100)	-	54 (100)	-	54 (100)	-
ESBLs + NDM (44)	-	44 (100)	-	44 (100)	-	44 (100)
NDM (33)	-	33 (100)	-	33 (100)	-	33 (100)
OXA (1)	-	1 (100)	1 (100)	-	-	1 (100)
OXA + CTX-M (9)	4 (44.44)	5 (55.55)	2 (22.22)	7 (77.77)	2 (22.22)	7 (77.77)
OXA + NDM (4)	-	4 (100)	-	4 (100)	-	4 (100)
KPC + SHV (1)	1 (100)			1 (100)		1 (100)
KPC (4)	4 (100)	-		4 (100)	-	4 (100)
Total	134	87	128	93	127	94

**Table 4:** Comparison of susceptibility pattern to non-β-lactam antibiotic in non-ESBL, ESBL producers, and carbapenem-resistant (CR) and carbapenem-sensitive (CS) isolates.

Antibiotic	Non ESBL *n* = 65 (%)	ESBL *n* = 125 (%)	*p* Value	CS *n* = 190 (%)	CR *n* = 99 (%)	*p* Value *
Ampicillin	65 (100)	97 (77.6)	<0.0001	162 (85.26)	24 (24.24)	<0.0001
Fosfomycin	65 (100)	86 (68.8)	<0.0001	115 (60.52)	43 (43.43)	<0.0001
Norfloxacine	59 (90.76)	37 (12.80)	<0.0001	96 (50.52)	1 (1.01)	<0.0001
Nitrofurantoin	60 (92.30)	85 (68)	<0.0001	145 (76.31)	35 (35.35)	<0.0001
Ciprofloxacin	59 (90.76)	38 (30.4)	<0.0001	97 (51.05)	3 (3.03)	<0.0001
Trimethoprim/sulfamethoxazole	51 (78.46)	21 (16.8)	<0.0001	72 (37.89)	3 (3.03)	<0.0001
Nalidixic acid	57 (87.69)	27 (21.6)	<0.0001	84 (44.21)	1 (1.01)	<0.0001

* The results are significant at *p* < 0.05.

## Data Availability

Not applicable.

## Supplementary Materials

The following supporting information can be downloaded at: https://www.mdpi.com/article/10.3390/antibiotics12101481/s1, Table S1: Details of primers, amplicon size and annealing temperature of the primers used for the genotypic detection of ESBLs and carbapenemase genes; Figure S1: Phenotypic detection of MBL and KPC; Figure S2: *bla*_NDM_ amplicon (984 bp); Figure S3: *bla*_OXA-48_ amplicon (281 bp).
