# Supplementary material for: In Vitro Activity of New β-Lactamase Inhibitor Combinations against blaNDM, blaKPC, and ESBL-Producing Enterobacteriales Uropathogens
Source: Antibiotics (Basel). 2023 Sep 25;12(10):1481. doi: 10.3390/antibiotics12101481 (PMC10604030; doi:10.3390/antibiotics12101481)
Supplement: Supplementary file 1 [file antibiotics-12-01481-s001.zip › antibiotics-2602965-supplementary.pdf]

Table S1. Details of primers, amplicon size and annealing temperature of the primers used for the genotypic detection of ESBLs and carbapenemase genes

Genes	Sequence (5to 3)	Amplicon size bp	Temp. °C	Reference
bla$_{TEM}$	F: TCGGGGAAATGTGCG<br>R:TGCTTAATCAGTGAGGCACC	971	59.2	[21]
bla$_{SHV}$	F:GCCGGGTTATTCTTATTTGTCGC<br>R: ATGCCGCCGCCAGTCA	1,007	62	[21]
bla$_{CTX-M-4}$	F:TTAATGATGACTCAGAGCATTC<br>R: GATACCTCGCTCCATTTATTG	901	55.4	[22]
bla$_{NDM}$	F: CACCTCATGTTTGAATTCGCC<br>R: CTCTGTCACATCGAAATCGC	984	64	[28]
bla$_{KPC}$	F: CTTGTCTCTCATGGCCGCTGG<br>R:ACGGAACGTGGTATCGCCGAT	449	55	[45]
bla$_{OXA-48}$	F: GCTTGATCGCCCTCGATT<br>R:GATTTGCTCCGTGGCCGAAA	281	60	[46]
bla$_{IMP}$	F: GGAATAGAGTGGCTTAATTC<br>R: CAACCAGTTTTGCCTTACC	327	52	[28]
bla$_{VIM}$	F: GGTGTTTGGTCGCATATCGC<br>R: CCATTCAGCCAGATCGGCATC	503	58	[28]

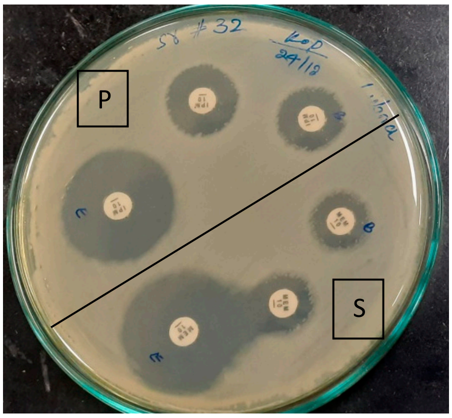

Fig S1. Phenotypic detection of MBL and KPC P: positive control, S: tested isolate

E: EDTA, B: phenylboronic acid (PBA). Both isolates were positive for MBL and negative for KPC.

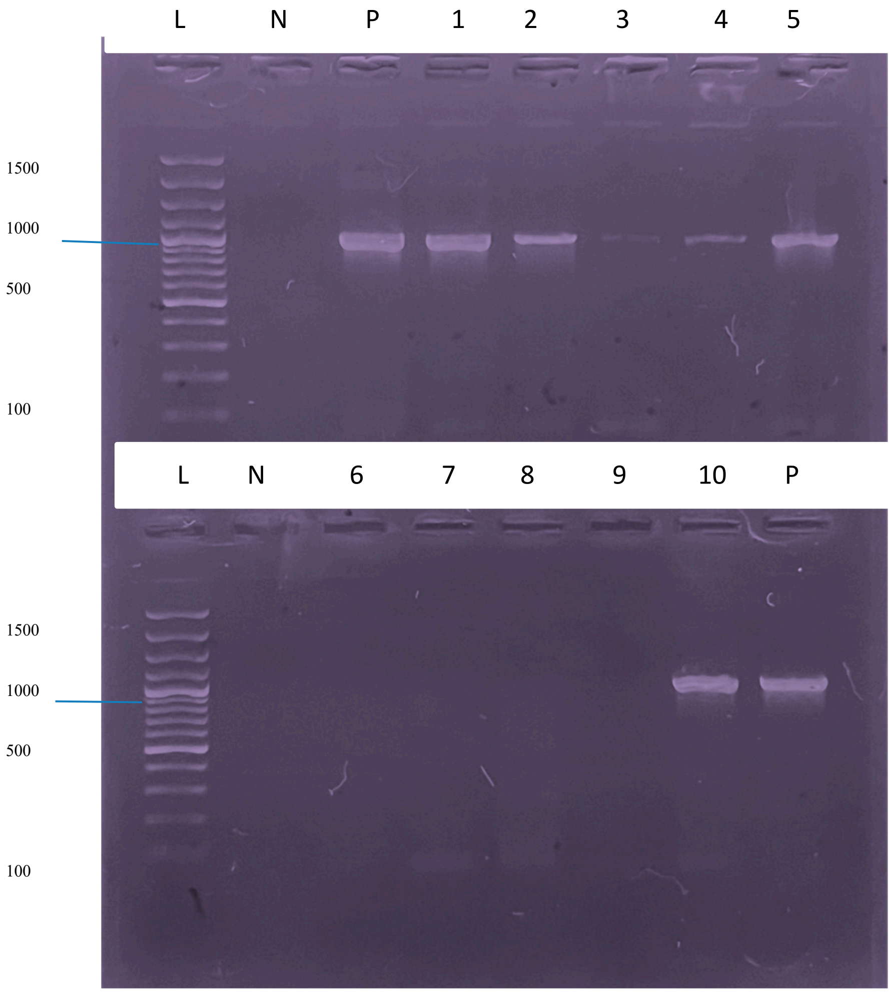

Fig S2. *bla*$_{NDM}$ amplicon (984bp). L: ladder, N: negative control, P: positive control.

The samples # 1,2,3,4,5 and 10 were positive for *bla*$_{NDM.}$ Sample # 3 disply a week band, sample was repeated and were positive.

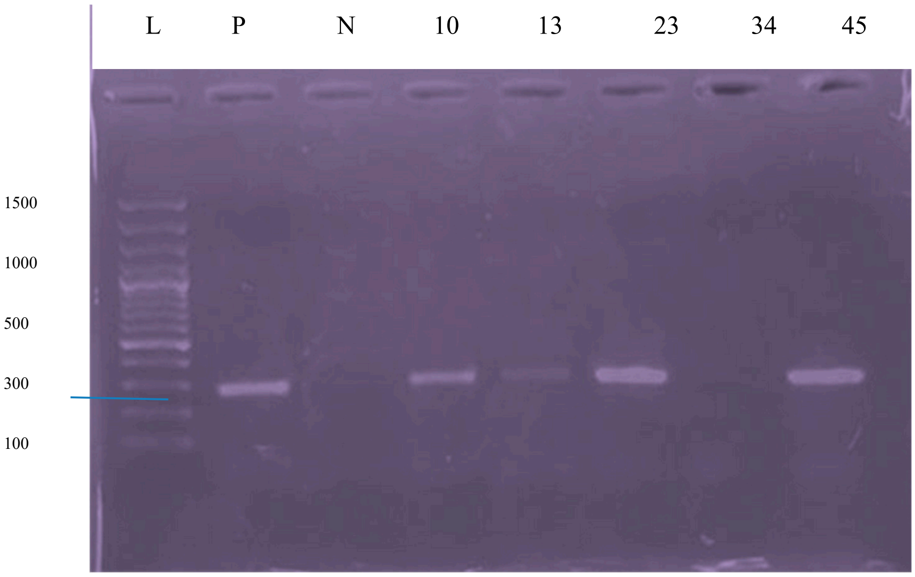

Fig S3. *bla*$_{OXA-48}$ amplicon (281bp). L: ladder, N: negative control, P: positive control.

The samples # 10, 13, 23, and 45 were positive for *bla*$_{OXA-48.}$
